# Gardenia oil increases estradiol levels and bone material density by a mechanism associated with upregulation of COX-2 expression in an ovariectomized rat model

**DOI:** 10.3892/etm.2013.1168

**Published:** 2013-06-19

**Authors:** BAOLI LI, YONGLI ZHANG, BINGYIN SHI, YAHUI CHEN, ZHENGXIANG ZHANG, TAO LIU

**Affiliations:** 1 Department of Pharmacology, Medical College, Yan’an University;; 2 Department of General Medicine, Cardiovascular Hospital of Yan’an University, Shaanxi 716000;; 3 Department of Endocrinology, First Affiliated Hospital of Xi’an Jiaotong University, Xi’an 710061;; 4 Department of Physiology, Medical College, Yan’an University, Xi’an, Shaanxi 716000, P.R. China

**Keywords:** gardenia oil, hormone, bone material density, bone biomechanics

## Abstract

This study aimed to determine the effects and mechanisms of gardenia oil on bone density and bone biomechanics in ovariectomized female rats. An ovariectomized rat model was established and the rats were administered various doses of gardenia oil. Rats administered diethylstilbestrol or saline served as the positive and the untreated controls, respectively. All rats received the same surgery, with the exception of the ovariectomy in the sham group. The levels of serum 17β-estradiol, follicle-stimulating hormone, luteinizing hormone, alkaline phosphatase (ALP) and calcium, and the bone material density (BMD), maximum stress and maximum strain were determined. The expression of COX-2 was also determined by immunoblotting and quantitative PCR (qPCR). Gardenia oil increased the serum levels of 17β-estradiol, the BMD, and the maximum stress and maximum strain of bones. The levels of COX-2 protein and COX-2 mRNA were significantly increased in the gardenia oil-treated rats. In conclusion, gardenia oil increases estradiol levels and BMD in an ovariectomized rat model. The effects of gardenia oil are associated with upregulation of the expression of COX-2.

## Introduction

Phytoestrogens, including isoflavones, lignans and coumestans, are components of plants and seeds (1). The molecular structures of phytoestrogens and mammalian estrogens are similar. Phytoestrogens aid the cure of hormone-associated diseases, including breast cancer, prostate cancer, menopausal syndromes, cardiovascular diseases and osteoporosis (2–6).

Due to the reduction in ovarian function for women during their perimenopausal stages, estrogen levels are often decreased, leading to secondary hyperparathyroidism and reduced calcitonin secretion. Therefore, bone resorption rates are often greater than bone formation rates, resulting in osteoporosis. For women with osteoporosis, bone material density (BMD) is decreased and the bone microstructures begin to degenerate. Thus, the bones become fragile and may easily fracture (7). Estrogen supplements may attenuate the long-term lack of estrogen caused by atrophic diseases of the genitourinary system, skin and brain. Supplements may also prevent osteoporosis, fractures and cardiovascular and cerebrovascular diseases. Bone estrogen promotes increased bone density and the length of the bone (8,9). Estrogen is involved in the formation of bone; when estrogen levels decrease, a gradual loss of calcium in the bones occurs. Osteocalcin loss results in osteoporosis.

Hormone replacement therapy is often used clinically. However, the application of hormone replacement therapy is limited due to numerous contraindications and adverse responses. The gardenia oil is extracted from seeds of the plant *Gardenia jasminoides*. Gardenia oil has been observed to affect the learning ability and memory of mice (10–12). COX-2 is a protein associated with the functions of estrogen in numerous diseases (13,14). In the current study, gardenia oil was used to treat ovariectomized rats. The effects of gardenia oil on 17β-estradiol (also termed E_2_) levels, BMD and bone biomechanics in ovariectomized female rats were investigated. Quantitative PCR (qPCR) and immunoblot assays were performed to study the associated molecular mechanisms. We observed that gardenia oil significantly increases the expression levels of COX-2 protein by increasing the mRNA levels.

## Materials and methods

### Experimental animals

SD rats with the average age of 3 months and weight of 280±20 g were provided by the experimental animal center, Medical College, Xi’an Jiaotong University (Xi’an, China). The SD rats (n=53) were divided into 6 groups (sham group, n=10; untreated group, n=12; diethylstilbestrol group, n=9; high dose group, n=7; middle dose group, n=7; low dose group, n=8). All animal experiments were conducted according to the ethical guidelines of Yan’an University (Xi’an, China).

The ovariectomized animal models were established as follows. Sodium pentobarbital anesthesia (3%) was administered to the healthy female SD rats (1 ml/kg) by intraperitoneal injection and ovariectomy surgery was performed. Following surgery, 400,000 units of penicillin was intramuscularly injected daily for 3 days to prevent infection. The rats receiving ovariectomies were randomly divided into the following groups: the high (4.50 g/kg), middle (1.80 g/kg) and low (0.72 g/kg) dose gardenia oil groups, the diethylstilbestrol group and the untreated control group. The rats treated with diethylstilbestrol (the diethylstilbestrol group) or saline (the untreated control group) served as the positive and the untreated controls, respectively. In addition to the five groups described above, the sham group served as the false-surgery control, since the rats in this group received the same surgery as others, with the exception of the ovariectomy. Fat of the same volume as the ovaries was removed from the sham group. The rats in the sham group were also administered saline.

The control and sham-operated groups were medicated with an equal volume of saline. After 12 weeks of administration and 12 h of fasting, the anesthetized animals were sampled.

### Experimental drug

The gardenia oil was provided by Xi’an Dongsheng Pharmaceutical Research Institute (Xi’an, China). Diethylstilbestrol, estrogen, follicle-stimulating hormone and luteinizing hormone were determined by electrochemical immunoassay. The diagnostic reagents, standards and quality controls were provided by Roche (Basel, Switzerland). Detection kits for serum alkaline phosphatase (ALP), calcium and phosphorus were purchased from Nanjing Jiancheng Bioengineering Company (Nanjing, China).

### Experimental equipment

The DEXA Dual Energy X-ray Absorptiometry equipment (Hologic QDR, 2000 type) was purchased from Hologic (Bedford, MA, USA). The fully automated chemiluminescence immunoassay analyzer COBASE411 and the SWD-10 microcomputer control electronic bone biomechanical analyzer were purchased from Roche.

### Blood collection and testing

After weighing, 3% sodium pentobarbital anesthesia (1 ml/kg) was administered to rats by intraperitoneal injection. Blood (3 ml) was collected via a carotid artery catheter. The levels of serum ALP, calcium and phosphorus were determined. Determination of 17β-estrogen, follicle-stimulating hormone and luteinizing hormone levels was also performed.

### Determination of BMD

Prior to the treatment, rat bone samples were collected. A dual-energy X-ray absorptiometry scan was performed. In order to ensure the quality of the scan, the Hologic QDR 2000 automatic internal quality control system was applied to maintain the precision to 0.4%.

### Determination of bone mechanics

Following measurement of the bone density of left femur specimens, a femoral three-point bending test was performed to determine the maximum stress and maximum strain. The space between two force points (L)=20 mm. The bending force (F) was measured. Two femoral central diameters, which were perpendicular, were selected as d1 and d2. The average diameter (d) was calculated. The maximum stress relative to the bending strength (ultimate strength) was calculated using the formula: σ=My/L=(8 LF)/(πd^3^). Bending moment on the section (M) and the distance to the neutral axis (y) were used. The load measurement accuracy was 0.1 N. The maximum strain measurement accuracy was 0.001 mm.

### Quantitative (qPCR)

The ovary-adjacent tissues of the rats were collected for RNA isolation using the RNeasy FFPE kit (Cat. no., 73504, Qiagen, Valencia, CA, USA). qPCR analysis of COX-2 mRNA and GAPDH mRNA levels was performed. The RT-PCR experiments were repeated at least 3 times. RNA was reverse transcribed into cDNA using random primers in a Reverse Transcription II system (Promega, Madison, WI, USA) according to the manufacturer’s instructions. Expression of COX-2 mRNAs was quantified by qPCR using an ABI Prism Sequence Detection system (Applied Biosystems, Foster City, CA, USA). An assay reagent containing premixed primers and a VIC-labeled probe (Applied Biosystems; cat. no. 4310884E) was used to quantify expression of endogenous GAPDH mRNA. The levels (mean value) of COX-2 mRNA transcripts were calculated. The COX-2 upstream and downstream primers were 5′-CGGGATCCTGCCAGCTCCACCG and 5′-GCTCTAGAACAAACTGAGTGAGTCC, respectively. The GAPDH upstream and downstream primer sequences were 5′-TGAAGGTCGGAGTCAACGGATTTGGT and 5′-CATGTGGGCCATGAGGTCCACCAC, respectively.

### Immunoblot assays

The total proteins were harvested from the ovary-adjacent tissues of the rats. The proteins were separated on 10% SDS/PAGE gels and then subjected to immunoblot analyses. The primary antibodies against COX-2 (∼70 kDa) and β-actin were purchased from Santa Cruz Biotechnology, Inc. (Santa Cruz, CA, USA; anti-COX-2, cat. no. sc-70879, 1:200; anti-β-actin, cat. no. sc-130301, 1:10,000). The secondary antibodies used in this study were goat anti-mouse IgG-HRP (cat. no. sc-2005, 1:10,000, Santa Cruz). The bound antibodies were detected using the ECL system (Pierce Biotechnology, Rockford, IL, USA). The immunoblot experiments were repeated at least 3 times.

### Statistical analysis

Each set of data was expressed as mean ± SEM. Significance was analyzed using SPSS 16.0 statistical analysis software (SPSS, Inc., Chicago, IL, USA) for one-way ANOVA and Newman-Keuls comparison test. P<0.01 and P<0.05 were considered to indicate a statistically significant difference.

## Results

### Gardenia oil increases 17β-estradiol levels in ovariectomized female rats

To determine if the gardenia oil affects 17β-estradiol levels in the ovariectomized female rats, the ovariectomized rats were administered various doses of gardenia oil (high dose group, 4.50 g/kg gardenia oil; middle dose group, 1.80 g/kg gardenia oil; low dose group, 0.72 g/kg gardenia oil). The rats administered diethylstilbestrol or saline served as the positive and the untreated controls, respectively. The sham group served as the false-surgery control, since the rats in this group received the same surgery, with the exception of the ovariectomy. Fat of the same volume as the ovaries was removed from the sham group. The rats in the sham group were also medicated with saline.

**Figure 1.**
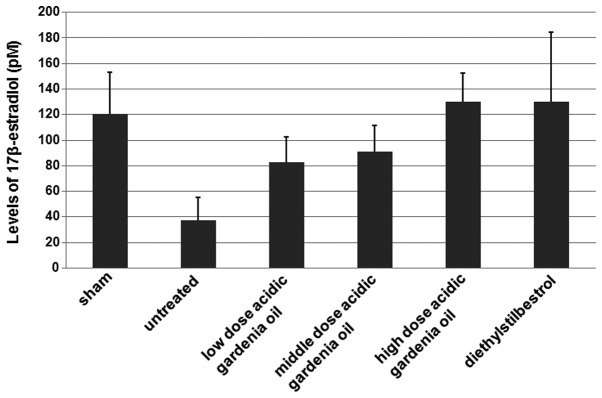
Levels of 17β-estradiol in the rats. The ovariectomized rats were administered various doses of gardenia oil (high dose group, 4.50 g/kg gardenia oil; middle dose group, 1.80 g/kg gardenia oil; low dose group, 0.72 g/kg gardenia oil). The rats administered diethylstilbestrol or saline served as the positive and the untreated controls, respectively. The sham group served as the false-surgery control. The rats in the sham group were also treated with saline. Blood (3 ml) was collected via the carotid artery catheter from the rats and the levels of 17β-estrogen were measured.

As shown in Fig. 1, the 17β-estradiol levels in the untreated group were decreased in comparison with those in the sham group where the rats did not receive the ovariectomy. When compared with the untreated control, the ovariectomized female rats in the gardenia oil groups had increased levels of 17β-estradiol. The 17β-estradiol levels in the high dose gardenia oil group were similar to the 17β-estradiol levels in the diethylstilbestrol group (positive control). These results suggest that gardenia oil increases the levels of 17β-estradiol in the ovariectomized female rats.

Follicle-stimulating hormone and luteinizing hormone are two hormones associated with 17β-estradiol. As listed in Table I, the gardenia oil significantly reduced the follicle-stimulating hormone and luteinizing hormone levels in ovariectomized female rats compared with the levels in the untreated group. These results suggest that gardenia oil has similar effects on follicle-stimulating hormone and luteinizing hormone levels as diethylstilbestrol.

In the ovariectomized rats, the gardenia oil reduced the levels of the serum ALP and elevated the levels of calcium and phosphorus compared with those in the untreated control group (Table II). The levels of the serum ALP were also reduced and the levels of calcium and phosphorus were also increased in the diethylstilbestrol group (Table II). These pieces of evidence also indicated that gardenia oil has a similar effect to diethylstilbestrol.

### Gardenia oil significantly increases BMD and the maximum stress and maximum strain of bone

In the untreated ovariectomized female rats, the BMD and the bone maximum stress and maximum strain were decreased when compared with those of the sham group (Table III). Diethylstilbestrol increased the BMD and the bone maximum stress and maximum strain compared with those in the untreated control group (Table III). Gardenia oil also increased the BMD and bone maximum stress and maximum strain compared with those in the untreated control group. These results indicate that gardenia oil has a similar effect to diethylstilbestrol on BMD and the maximum stress and the maximum strain of bone.

### Levels of COX-2 protein are significantly increased in the high dose gardenia oil group

**Figure 2.**
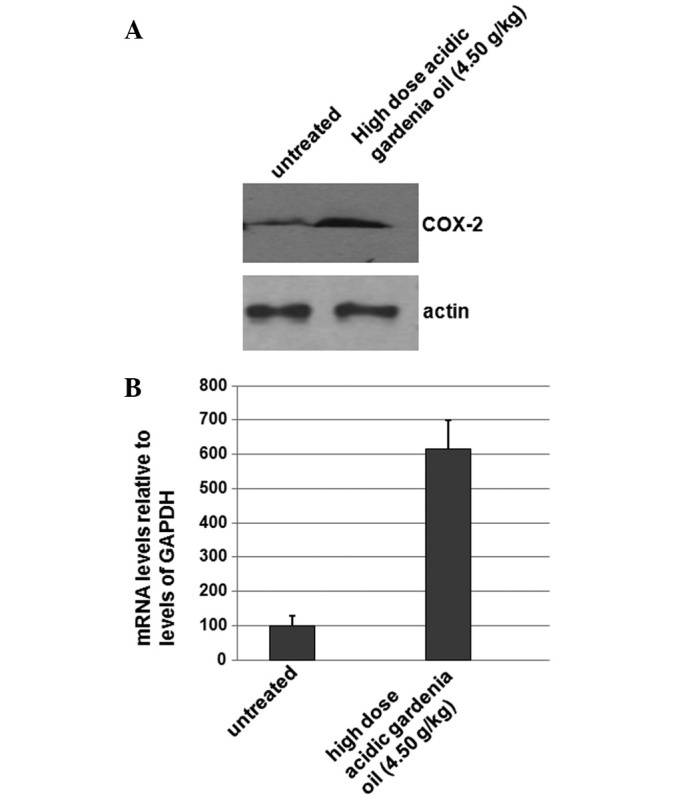
Expression levels of COX-2 in the untreated control group and the high dose gardenia oil group. (A) Immunoblots of COX-2 expression. The total proteins were harvested and subjected to immunoblot analyses. The experiments were repeated at least three times. (B) Quantitative PCR (qPCR) analysis of COX-2 mRNA levels in the rats. The total RNAs were harvested from tissues. The RT-PCR experiments were repeated at least 3 times. RNA was reverse transcribed into cDNA using random primers in a Reverse Transcription II system (Promega) according to the manufacturer’s instructions. The mRNAs were quantified by qPCR using an ABI Prism Sequence Detection System (Applied Biosystems). Template-negative and RT-negative conditions were used as controls. Amplification of the endogenous GAPDH cDNA was monitored. The levels (mean value) of COX-2 relative to those of GAPDH transcripts were calculated.

To determine if gardenia oil affects the expression levels of COX-2, the total proteins were isolated from the untreated control group and the gardenia oil group. Western blotting was performed; as shown in Fig. 2A, the expression levels of COX-2 were significantly increased in the gardenia oil group when compared with the levels in the untreated control group. The levels of β-actin were used as a loading control. The results shown in Fig. 2A indicate that the expression levels of COX-2 were increased in the gardenia oil group, which may be associated with its effects on BMD.

### Levels of COX-2 mRNA are significantly increased in the high dose gardenia oil treatment group

To determine if gardenia oil affects the mRNA levels of *COX-2* gene, the total RNAs were isolated from the untreated control and the gardenia oil group. qPCR was performed to detect the levels of *COX-2* mRNA. As shown in Fig. 2B, the mRNA levels of *COX-2* were significantly increased ∼6-fold in the gardenia oil group when compared with the levels in the untreated control group. The results shown in Fig. 2B suggest that gardenia oil may increase levels of *COX-2* mRNA.

## Discussion

In this study, through the determination of bone density and bone biomechanics, it was observed that gardenia oil increases BMD and the maximum stress and maximum strain of bones in ovariectomized female rats. BMD and the maximum stress and maximum strain of bones are closely associated with the quality and microstructural integrity of bones and osteoporosis.

In the prevention and treatment of osteoporosis, medicines derived from plants have been widely used because of their relatively minor side-effects. Gardenia oil contains oleic acid, linoleic acid and linolenic acid (10). Oleic acid and linoleic acid may generate γ-linolenic acid, arachidonic acid and prostaglandins. The α-linolenic acid exists mainly as EPA and DHA in the body (15,16). In the current study, we demonstrated that gardenia oil may increase estrogen levels due to its effects on COX-2 expression. Gardenia oil reduces the serum ALP level and increases levels of calcium and phosphorus, thereby attenuating osteoporosis. It has been reported that ω-3 polyunsaturated fatty acids derived from oleic acids, linoleic acids and linolenic acids may reduce the rate of postmenopausal bone loss (17–19).

The mechanism of action of gardenia oil in the regulation of estrogen levels is unclear. However, in the current study, we observed that the mRNA levels of *COX-2* were significantly increased ∼6-fold in the gardenia oil group when compared with the levels in the untreated control group. The results indicate that gardenia oil increases the levels of *COX-2* mRNA. The expression levels of COX-2 protein were significantly increased in the high dose gardenia oil group when compared with the levels in the untreated control group. These results demonstrate that the expression level of COX-2 was increased in the gardenia oil group, which may be associated with its effects on BMD. By increasing the level of COX-2 expression, gardenia oil may induce metabolic changes, such as promoting the ovaries and other glands to secrete estrogen (20).

## Figures and Tables

**Table I.** Effects of gardenia oil on sex hormones of ovariectomized female rats (mean ± SEM).

Group	Number of rats	Follicle-stimulating hormone (U/l)	Luteinizing hormone (U/l)
Sham	10	0.25±0.07^b^	0.24±0.06^a^
Untreated	12	0.35±0.16	0.44±0.17
Diethylstilbestrol	9	0.06±0.04^a^	0.20±0.07^a^
High dose	7	0.22±0.07^b^	0.27±0.08^a^
Middle dose	7	0.26±0.07	0.32±0.07^b^
Low dose	8	0.27±0.06	0.34±0.10

a P<0.01,

b P<0.05 vs. model group.

**Table II.** Effects of gardenia oil on the serum alkaline phosphatase, calcium and phosphorus levels of ovariectomized female rats (mean ± SEM).

Group	Number of rats	ALP (pg/ml)	Ca (mM)	P (mmol/l)
Sham	10	143.50±47.29	2.44±0.13	2.34±0.21
Untreated	12	147.17±15.11	2.40±0.16	2.29±0.19
Diethylstilbestrol	9	91.80±22.15^a^	2.56±0.16^b^	2.48±0.19^b^
High dose	7	104.57±26.55^a^	2.66±0.17^a^	2.49±0.28^b^
Middle dose	7	116.33±24.71^b^	2.48±0.12	2.35±0.22
Low dose	8	127.83±29.41^a^	2.45±0.15	2.30±0.20

a P<0.01,

b P<0.05 vs. model group. ALP, alkaline phosphatase.

**Table III.** Effects of acid parker gardenia oil on bone material density and bone biomechanics of ovariectomized female rats (mean ± SEM).

Group	Number of rats	Tibiofibular BMD (g/cm^2^)	Thigh BMD (g/cm^2^)	Thigh maximum stress (N)	Thigh maximum strain (mm)
Sham	10	0.145±0.015	0.146±0.026	132.4±23.7	1.32±0.43
Untreated	12	0.142±0.014	0.145±0.021	130.4±23.1	1.08±0.12
Diethylstilbestrol	9	0.171±0.043^b^	0.186±0.051^b^	155.8±16.2^a^	1.77±0.27^a^
High dose	7	0.163±0.031^b^	0.157±0.021^b^	143.0±15.8^b^	1.48±0.25^a^
Middle dose	7	0.146±0.016	0.170±0.040^b^	147.3±37.0	1.45±0.29^a^
Low dose	8	0.152±0.024	0.149±0.025	142.5±21.7	1.14±0.35

a P<0.01,

b P<0.05 vs. model group. BMD, bone material density.
